# Assessment of Donor-Site Morbidity Using Shear Wave Elastography After Peroneus Longus Autograft Harvest for ACL Reconstruction

**DOI:** 10.3390/jcm15124473

**Published:** 2026-06-09

**Authors:** Mehmet Boz, Gülseda Boz, Mehmet Akcicek, A. Alper Şahin, Tarık Altunkılıç, Bünyamin Arı, İsmail Güzel, Murat Can

**Affiliations:** 1 Department of Orthopedics and Traumatology, Faculty of Medicine, Malatya Turgut Ozal University, Malatya 44210, Türkiye; 2 Department of Public Health, Faculty of Medicine, Inonu University, Malatya 44280, Türkiye; gulseda.boz@inonu.edu.tr; 3 Department of Radiology, Faculty of Medicine, Malatya Turgut Ozal University, Malatya 44210, Türkiye; 4 Department of Orthopaedic and Traumatology, Ordu University Training and Research Hospital, Ordu 52200, Türkiye; 5 Department of Orthopedics and Traumatology, Malatya Training and Research Hospital, Malatya 44330, Türkiye

**Keywords:** anterior cruciate ligament reconstruction, peroneus longus tendon, autograft, donor-site morbidity, shear wave elastography

## Abstract

**Background/Objectives**: The peroneus longus tendon has gained attention as an alternative autograft source for anterior cruciate ligament reconstruction; however, donor-site morbidity remains a concern because of its role in ankle eversion and lateral ankle function. **Methods**: This cross-sectional observational study evaluated whether peroneus longus tendon harvest causes measurable donor-site changes using shear wave elastography. Forty patients who underwent anterior cruciate ligament reconstruction with a peroneus longus tendon autograft and who completed at least 12 months of follow-up were included. The graft-harvested side was compared with the contralateral side in terms of ankle plantar flexion, eversion range of motion, and shear wave elastography-derived tendon stiffness. **Results**: Pain was assessed using the visual analog scale. Postoperative VAS scores were significantly lower than preoperative scores [8 (5–9) vs. 2 (1–4); *p* < 0.001]. No significant differences were found between the graft-harvested and contralateral sides in plantar flexion, eversion, or tendon stiffness. A strong positive correlation was observed between operated-side and contralateral-side shear wave elastography values (r = 0.950; *p* < 0.001). **Conclusions**: Peroneus longus tendon harvest was not associated with measurable residual tendon stiffness or ankle range-of-motion changes at 12 months.

## 1. Introduction

Anterior cruciate ligament (ACL) injury is among the most common knee joint injuries encountered in sports trauma, and may result in chronic knee instability, secondary meniscal injury, and premature osteoarthritic degeneration when left untreated [1,2]. In young, physically active patients, arthroscopic anterior cruciate ligament reconstruction (ACLR) is widely regarded as the current gold standard for treating ACL injury [3], with its main objective being to reestablish a stable knee joint that allows patients to resume normal daily activities and return to sports after surgery [4]. Graft choice in ACLR has changed substantially over the past few decades, and auto-, allo-, and synthetic grafts have been investigated with varying levels of clinical success [5]. Graft selection is a critical determinant of ACLR outcomes, yet the optimal graft source remains a matter of ongoing debate; an ideal graft should replicate the complex anatomy of the native ACL, possess biomechanical properties comparable to those of the intact ligament, allow for strong and reliable fixation, promote rapid biological incorporation, and minimize donor-site morbidity [6]. Among the available options, autografts remain the preferred choice in ACL surgery, with bone–patellar tendon–bone and hamstring tendon grafts being the most commonly used [7]. Given the disadvantages of hamstring tendon (HT) grafts, alternative autograft sources with lower donor-site morbidity remain elusive. Moreover, the increasing frequency of multiligament reconstructions has further highlighted the need for additional graft options. The peroneus longus (PLT) autograft has recently attracted attention as a potential alternative because of its favorable characteristics, including a greater graft diameter, high ultimate load to failure, and relative ease of harvest [8]. Nevertheless, the available literature on clinical outcomes and donor-site morbidity remains scarce. Since the PLT is a key contributor to ankle eversion, concerns persist regarding possible ankle function impairment and postoperative instability following graft harvest [9]. Ultrasound elastography (UE) has emerged as a valuable modality in musculoskeletal imaging, offering quantitative assessment of tissue mechanical properties under both physiological and pathological conditions [10,11]. By complementing conventional ultrasonography with information on tissue stiffness, UE enhances diagnostic utility across a range of soft-tissue pathologies [10,11,12,13]. Among its modalities, shear wave elastography (SWE)—first described by Sarvazyan et al. in 1998 [14]—generates shear waves via an acoustic radiation force impulse (ARFI) and quantifies tissue stiffness in meters per second (m/s) or, via mathematical conversion, as Young’s modulus in kilopascals (kPa) [10,13,14]. Encompassing both strain and shear wave techniques [10,15], SWE has demonstrated growing applicability in tendons, muscles, ligaments, and peripheral nerves and may offer practical advantages over MRI in terms of accessibility and cost [11,13,16,17]. Given the limited evidence regarding donor-site morbidity after PLT harvest, in the present study, we aimed to determine whether using the peroneus longus tendon as an autograft in ACL reconstruction results in measurable changes in the donor site, as assessed by ultrasound elastography, by comparing the operated and contralateral sides.

## 2. Materials and Methods

### 2.1. Study Design and Patient Population

**Figure 1:**
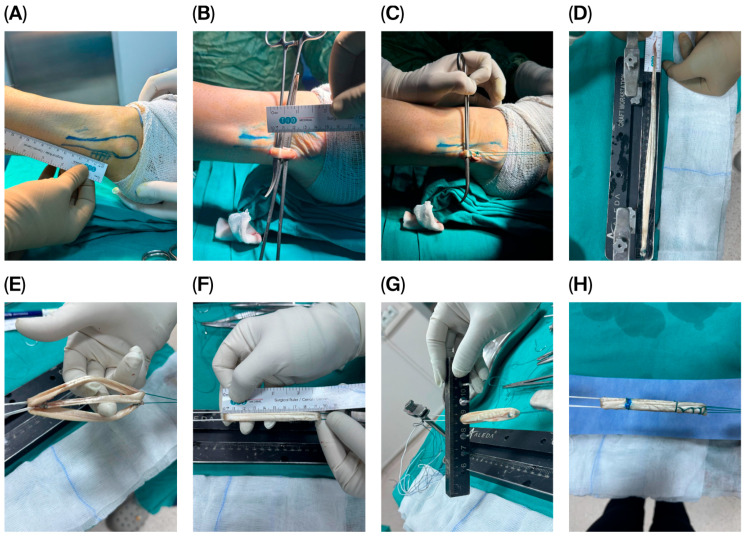
Intraoperative stages of peroneus longus tendon graft harvesting and preparation (**A**) Preoperative surface marking of the peroneus longus tendon harvest site. (**B**) Skin incision over the marked harvest area. (**C**) Identification and exposure of the peroneus longus tendon. (**D**) Isolation of the tendon from surrounding soft tissues. (**E**) Harvesting of the peroneus longus tendon graft. (**F**,**G**) Measurement of graft length and diameter. (**H**) Final prepared peroneus longus tendon autograft ready for implantation.

In this cross-sectional observational study, we included 40 patients who underwent unilateral anterior cruciate ligament reconstruction using a peroneus longus tendon autograft between January 2024 and January 2025 and were evaluated after at least 12 months of postoperative follow-up. Clinical and surgical data were collected retrospectively from medical records. Ethics committee approval was obtained on 31 March 2026 before the initiation of the follow-up assessments. Following approval, participants were contacted and invited to a follow-up evaluation, and shear-wave elastography (SWE) examinations of both the donor and contralateral peroneal tendons were performed between 6 and 10 April 2026. A skin and subcutaneous incision of approximately 2 cm was made posterior to the distal fibula, approximately 2–3 cm proximal to the tip of the lateral malleolus. After careful soft-tissue dissection, the peroneus longus and peroneus brevis tendons were identified. The peroneus longus tendon was tenodesed to the peroneus brevis tendon at the level of the lateral malleolus. Subsequently, the peroneus longus tendon was transversely transected approximately 5 mm proximal to the tenodesis site. From this transection point, a full-thickness peroneus longus tendon graft was harvested proximally toward the musculotendinous junction using appropriate tendon-harvesting instruments. Care was taken throughout the procedure to protect the superficial peroneal nerve and surrounding soft tissues. (Figure 1). After harvesting, the graft was prepared using a tendon preparation instrument (Aleda Medical, Istanbul, Turkey). The graft-harvested side was compared with the contralateral side in terms of ankle function and tendon properties.

The inclusion criteria were patients who underwent unilateral ACL reconstruction using a peroneus longus tendon autograft, completed at least 12 months of postoperative follow-up, and had complete clinical, functional, and elastographic data. The exclusion criteria were previous surgery or trauma involving the ankle or foot, bilateral ACL injury, contralateral lower extremity pathology, systemic inflammatory disease, neuromuscular disorder, diabetes mellitus, chronic tendon disease, or incomplete follow-up data.

A priori power analysis was performed using G*Power software (version 3.1.9.7, Heinrich Heine University Düsseldorf, Düsseldorf, Germany) based on paired comparisons between the operated and contralateral sides. The primary outcome variable for sample size estimation was the difference in SWE-derived tendon stiffness between the graft-harvested and contralateral sides. Assuming an alpha level of 0.05 and a statistical power of 80%, the minimum required sample size was 34 patients; considering the potential for dropout and noncompliance, 40 patients were initially included in the study. Given that all the participants completed the study without being lost to follow-up, the final analysis was performed on all 40 patients.

### 2.2. Data Collection

Demographic data were recorded, including age, sex, height, weight, and body mass index (BMI). The dominant and operated sides were also documented. Shear wave elastography (SWE) was used to evaluate the mechanical properties of the peroneus longus tendon at the graft harvest site. In addition, ankle range of motion, including eversion and plantar flexion angles, was measured using a goniometer and recorded for analysis.

### 2.3. Clinical Assessment

Pain levels in the operated extremity were evaluated using the visual analog scale (VAS), a validated tool ranging from 0 (no pain) to 10 (worst imaginable pain). VAS1 represented the preoperative pain score of the operated side, whereas VAS2 represented the postoperative pain score of the same side at the final follow-up. All assessments were performed under standardized conditions during outpatient follow-up visits, and functional and elastographic parameters, including plantar flexion, eversion, and SWE values, were compared between the operated graft-harvested side and the contralateral healthy side.

### 2.4. Functional Assessment

**Figure 2:**
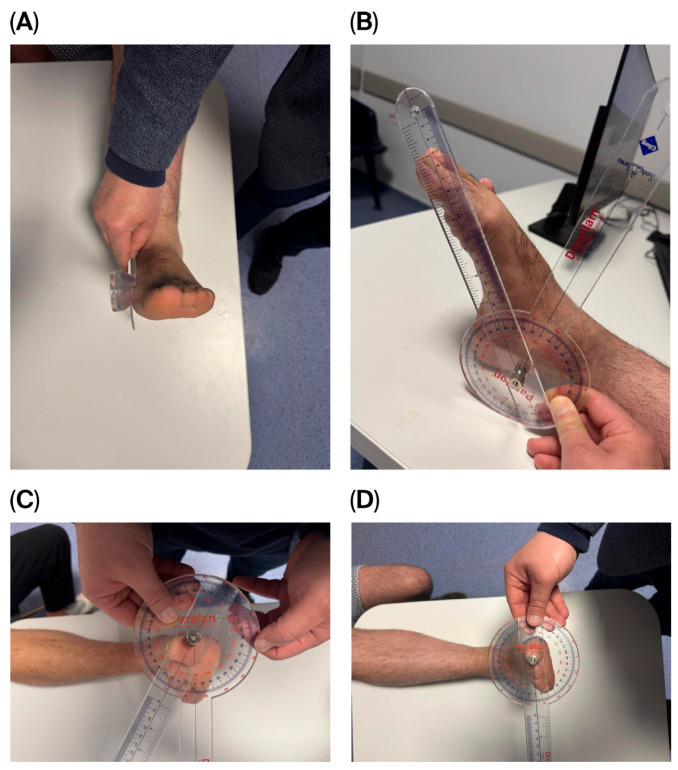
Goniometric measurement of ankle range of motion (plantar flexion and eversion). (**A**) Initial positioning for ankle range-of-motion assessment using a standard goniometer. (**B**) Measurement of ankle plantar flexion with the patient in the supine position and the knee maintained at 30° of flexion. (**C**) Measurement of ankle eversion using a standard goniometer with appropriate alignment of anatomical landmarks. (**D**) Final positioning during ankle eversion measurement from the neutral ankle position. Each measurement was repeated three times, and the mean value was recorded for analysis.

Ankle range of motion (ROM) was assessed using a standard goniometer. Plantar flexion and eversion measurements were performed with the patient in the supine position and the knee maintained at 30° of flexion, and all measurements initiated from a neutral ankle position. Each measurement was conducted three times by the same examiner, and the mean value was recorded for analysis to improve reliability and reduce intraobserver variability. Comparisons were made between the operated (graft-harvested) and the contralateral non-operated side (Figure 2).

### 2.5. Elastography Assessment

**Figure 3:**
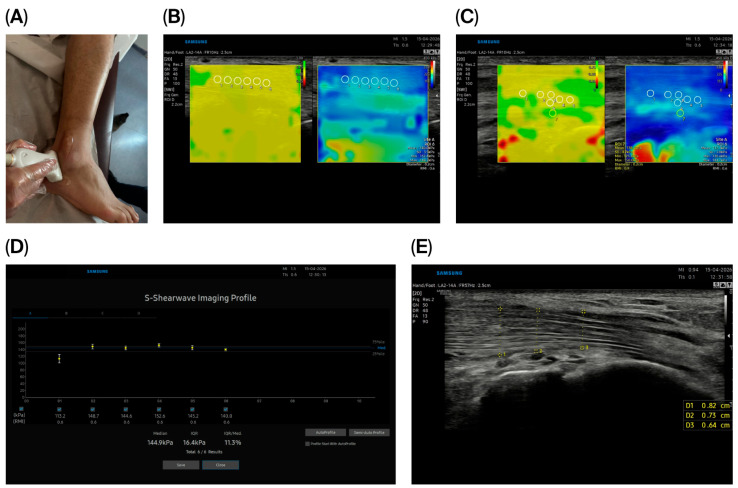
Shear wave elastography (SWE) evaluation of the peroneus longus tendon. (**A**) Probe placement during SWE examination with the patient in the lateral decubitus position. (**B**) Representative elastogram showing color-coded stiffness distribution with multiple regions of interest (ROIs) placed along the tendon. (**C**) Additional elastogram demonstrating variation in tendon stiffness values across different measurement points. (**D**) Shear wave imaging profile displaying quantitative stiffness measurements across selected ROIs. (**E**) Conventional B-mode ultrasound image illustrating tendon morphology and ROI placement. Elastograms are color-coded from blue (soft) to red (hard), and stiffness values are expressed in kilopascals (kPa). Measurements were obtained from multiple points, and the mean value was used for analysis.

Tendon elasticity was evaluated using shear wave elastography (SWE), with measurements obtained separately for the operated (graft-harvested) and the contralateral healthy side. All SWE measurements were performed by a radiologist with 12 years of experience to ensure consistency and reduce interobserver variability. During the examination, patients were positioned in the lateral decubitus position, and the ultrasound probe was placed longitudinally along the tendon. SWE values were expressed in kilopascals (kPa) and were accompanied by an automatically calculated reliability measurement index (RMI) to assess measurement consistency [18]. In accordance with previous studies, measurements with an RMI ≥ 0.8 and an interquartile range-to-median ratio (IQR/Med) ≤ 30% were considered reliable; therefore, these thresholds were adopted as quality criteria in this study. Elastography and stiffness measurements were performed using an ultrasound system (RS85 Prestige, Samsung Medison Co., Ltd., Seoul, Republic of Korea) equipped with a 12 MHz linear transducer, and the ROI size was fixed at 2 mm for all of the measurements. Measurements were obtained from six different points along the tendon, and the mean value was calculated for analysis (Figure 3).

### 2.6. Statistical Analysis

Statistical analyses were performed using IBM SPSS Statistics for Windows, version 22.0 (IBM Corp., Armonk, NY, USA). The normality of the data distribution was assessed using the Shapiro–Wilk test; variables with a normal distribution were expressed as the mean ± standard deviation, whereas those without were presented as the median (minimum–maximum). Comparisons were performed using the Wilcoxon signed-rank test and VAS scores were compared between the pre- and postoperative assessments of the operated limb, whereas plantar flexion, eversion, and SWE values were compared between the operated graft-harvested and contralateral healthy side. Correlations between variables were evaluated using Spearman’s correlation analysis, with effect size calculated using the formula r = Z/√N, where Z values were obtained from the Wilcoxon test. A *p* value of <0.05 was considered to indicate statistical significance.

### 2.7. Ethical Approval

This study was approved by the Malatya Turgut Özal University Clinical Research Ethics Committee (Approval No: 2026/154) and was conducted in accordance with the principles of the Declaration of Helsinki. Written and verbal informed consent was obtained from all participants prior to inclusion in the study.

## 3. Results

A total of 60% of the patients were aged ≤30 years, while 40% were ≥31 years; the mean age was 31.6 ± 5.9 years. The majority of the patients were male (95%, *n* = 38), and the mean body mass index was 26.3 ± 3.1 kg/m^2^ (range: 20.4–32.7). Since the VAS scores did not have a normal distribution, they are presented as medians (minimum–maximum): the median VAS1 and VAS2 scores were 8 (5–9) and 2 (1–4), respectively. The median range of motion for plantar flexion was 39° (32–48) on the operated and 38° (33–48) on the contralateral side; the median eversion range of motion was 21° (17–29) on the operated and 20° (17–28) on the contralateral side; and the median SWE values were 143.6 (85.8–163.5) kPa on the operated and 144.9 (86.1–160.6) kPa on the contralateral side (Table 1).

VAS scores of the operated limb significantly decreased from the pre- to postoperative period [VAS1: 8 (5–9) vs. VAS2: 2 (1–4); Z = −5.575; *p* < 0.001]. In contrast, no statistically significant differences were observed between the operated and contralateral sides in terms of plantar flexion [39° (32–48) vs. 38° (33–48); *p* = 0.875], eversion [21° (17–29) vs. 20° (17–28); *p* = 0.533], or SWE values [143.6 (85.8–163.5) kPa vs. 144.9 (86.1–160.6) kPa; *p* = 0.256] (Table 2).

Correlation analysis revealed strong and statistically significant negative correlations between postoperative VAS scores and SWE values measured on both the contralateral and operated sides. Specifically, postoperative VAS scores were negatively correlated with contralateral (r = −0.602; *p* < 0.001) and operated-side SWE values (r = −0.629; *p* < 0.001). In addition, strong positive correlations were observed between the operated and contralateral sides in terms of plantar flexion (r = 0.899; *p* < 0.001), eversion (r = 0.896; *p* < 0.001), and SWE values (r = 0.950; *p* < 0.001). A weak-to-moderate positive correlation was found between the contralateral SWE values and the operated-side plantar flexion (r = 0.336; *p* = 0.034), whereas no significant correlation was detected between the SWE values and the eversion parameters (*p* > 0.05). Effect size analysis demonstrated a large effect for VAS scores (r = 0.88), whereas plantar flexion, eversion, and SWE values showed negligible-to-small effect sizes. These correlation and effect size analyses are summarized in Supplementary Table S1.

## 4. Discussion

In the present study, we investigated donor-site morbidity following peroneus longus tendon harvest for ACL reconstruction using shear wave elastography as an objective quantitative assessment tool. The principal finding was that PLT harvest was not associated with significant alterations in residual tendon stiffness, as demonstrated by comparable SWE values between the operated and contralateral sides. In parallel, the plantar flexion and eversion range of motion were similar between the sides, indicating that ankle mobility was preserved after graft harvest. Postoperative pain scores were also significantly lower than preoperative values, suggesting satisfactory clinical recovery. Collectively, these findings indicate that PLT autograft harvest did not lead to measurable mechanical or functional deterioration at the donor site in this cohort.

The favorable donor-site profile of PLT harvest observed in the present study is broadly consistent with previous reports in the literature. Shi et al. reported that PLT autografts provide favorable graft characteristics and acceptable donor-site outcomes in ACL reconstruction, supporting their use as a viable alternative to conventional grafts [8]. A meta-analysis conducted by Kumar et al. demonstrated that peroneus longus and hamstring tendon autografts provide similar functional recovery and knee stability in anterior cruciate ligament reconstruction; however, the peroneus longus tendon offers additional advantages, such as a larger graft diameter and fewer donor-site complications [18]. However, many previous studies have relied mainly on subjective clinical assessments and conventional functional testing, with limited objective evaluation of residual tendon tissue properties. To the best of our knowledge, this is among the first studies to quantitatively evaluate the mechanical properties of the residual peroneus longus tendon after graft harvest using shear wave elastography. The absence of a significant difference in SWE values between the operated and contralateral tendons, together with the strong bilateral correlation (r = 0.950; *p* < 0.001), suggests that full-thickness peroneus longus tendon graft harvest does not result in measurable alterations in the elastographic properties of the residual tendon.

The SWE values recorded for both the operated and contralateral peroneus longus tendons in the present study (143.6 and 144.9 kPa, respectively) are consistent with reference values reported for healthy lower extremity tendons in the literature. Prior studies have reported mean SWE stiffness values of approximately 145.6 kPa for the Achilles tendon at its midportion in healthy individuals, with values for pathological tendons falling significantly lower—approximately 60 kPa—than those of healthy controls (185 kPa) across the Achilles, patellar, and epicondylar tendons [19,20]. The concordance between our findings and these established reference ranges further corroborates that the residual peroneus longus tendon maintains its mechanical integrity following full-thickness peroneus longus tendon graft harvest, with stiffness values within the range expected for structurally intact tendons. Shear wave elastography provides a quantitative, noninvasive method for evaluating tendon mechanical properties and has increasingly been used in musculoskeletal research to assess tendon stiffness and tissue quality. In the present study, SWE was used to evaluate the residual peroneus longus tendon following graft harvest objectively and demonstrated comparable stiffness values between the operated and contralateral sides. Beyond tendon stiffness evaluation, SWE may also have broader applications in postoperative musculoskeletal assessment. Su et al. reported the use of SWE to identify hardened scar tissue surrounding the radial nerve after humeral shaft fixation and to guide ultrasound-guided perineural hydrodissection in patients with postoperative pain and suspected nerve entrapment. These findings suggest that SWE may contribute not only to quantitative tendon evaluation but also to the assessment of postoperative soft-tissue alterations, scar-related stiffness, and interventional planning in complex musculoskeletal conditions [21].

In our study, the use of shear wave elastography as the primary assessment method provides an additional methodological advantage over other studies in the literature. In musculoskeletal applications, SWE has been shown to reliably detect mechanical changes in tendons under both physiological and pathological conditions, and its quantitative output—expressed in kilopascals—allows for direct bilateral comparisons with a level of precision that functional testing alone cannot provide [11,13]. In the present study, all SWE measurements were performed by a radiologist with 12 years of experience using a standardized protocol, with six measurement points obtained per tendon and mean values used for analysis, thereby minimizing intraobserver variability and enhancing the reliability of the data. From a clinical perspective, the combination of preserved ankle range of motion and comparable SWE values between sides suggests that PLT harvest is unlikely to result in clinically relevant donor-site impairment at the 12-month follow-up; however, the observed negative correlation between postoperative VAS scores and SWE values should be interpreted cautiously, as pain perception after ACL reconstruction is multifactorial and may not directly reflect donor-site tendon properties. Therefore, this correlation should be considered an exploratory finding rather than direct evidence of a causal relationship between tendon stiffness and postoperative pain. From a clinical perspective, the preservation of ankle range of motion together with comparable SWE-derived stiffness values between the operated and contralateral sides may assist surgeons in preoperative counseling, particularly for young and physically active patients. These findings also support using the peroneus longus tendon as a viable autograft option in revision ACL reconstruction or multiligament procedures where conventional graft sources may be limited.

## 5. Limitations

This study had several limitations. Firstly, the sample size was relatively small, and the study was conducted at a single center, which may have limited the generalizability of the findings. Secondly, the study population was predominantly male, thereby limiting the applicability of the results to female patients. Thirdly, although the contralateral limb served as an internal control, preoperative and serial postoperative SWE measurements were not available; therefore, baseline tendon stiffness and the temporal course of tendon remodeling after graft harvest could not be evaluated. Fourthly, ankle function was assessed by only using range-of-motion measurements, whereas objective muscle strength testing and validated foot–ankle functional outcome scores were not included. In addition, validated chronic ankle instability assessments, detailed proprioceptive evaluations, and donor-site-specific pain analyses were not systematically performed; therefore, subclinical lateral ankle instability and functional donor-site morbidity could not be fully excluded. Fifthly, all SWE measurements were performed by a single experienced radiologist, and interobserver reliability was not assessed. In addition, although SWE provides quantitative information on tendon stiffness, elastography measurements may be influenced by probe pressure, ankle position, tendon tension, and device-specific settings, despite the existence of standardized acquisition protocols. Finally, the follow-up period was limited to 12 months, and so longer-term studies are needed to determine whether these findings persist over time. Despite these limitations, in this study, we provide a standardized and quantitative elastographic evaluation of the residual peroneus longus tendon, contributing novel data to the limited literature and offering clinically relevant insight into donor-site assessment following ACL reconstruction.

In conclusion, peroneus longus tendon harvest for ACL reconstruction was not associated with measurable alterations in residual tendon stiffness or ankle range of motion at the 12-month follow-up. Comparable SWE-derived stiffness values and similar plantar flexion and eversion ranges of motion between the operated and contralateral sides suggest preserved mechanical and functional characteristics of the residual tendon. These findings support PLT as a viable autograft option, particularly when conventional graft sources are limited. However, further prospective studies with larger cohorts, objective strength testing, validated foot–ankle functional scores, and longer follow-up periods are needed to confirm the long-term donor-site safety of PLT harvest.

## Figures and Tables

**Table 1:** Demographic characteristics and clinical, functional, and elastography findings.

Variable	Value
Demographic Characteristics	
Age (years)	31.6 ± 5.9
≤30 years	24 (60%)
≥31 years	16 (40%)
Male	38 (95%)
Female	2 (5%)
BMI (kg/m^2^)	26.3 ± 3.1
Clinical Assessment	
VAS1 (Preoperative)	8 (5–9)
VAS2 (Postoperative)	2 (1–4)
Functional and Elastography Findings	
Plantar flexion (°)—Operated	39 (32–48)
Plantar flexion (°)—Contralateral	38 (33–48)
Eversion (°)—Operated	21 (17–29)
Eversion (°)—Contralateral	20 (17–28)
SWE—Operated (kPa)	143.6 (85.8–163.5)
SWE—Contralateral (kPa)	144.9 (86.1–160.6)

Data are presented as the mean ± standard deviation for normally distributed variables and median and minimum–maximum for non-normally distributed variables. Categorical variables are presented as numbers and percentages.

**Table 2:** Comparison of pain scores and bilateral functional and elastographic outcomes.

Variable	Comparison	Median (Min–Max)	Z Value	*p* Value
VAS	Preoperative VAS1	8 (5–9)	−5.575	<0.001
	Postoperative VAS2	2 (1–4)		
Plantar flexion	Operated side	39 (32–48)	−0.158	0.875
	Contralateral side	38 (33–48)		
Eversion	Operated side	21 (17–29)	−0.624	0.533
	Contralateral side	20 (17–28)		
SWE	Operated side	143.6 (85.8–163.5)	−1.137	0.256
	Contralateral side	144.9 (86.1–160.6)		

Data are presented as the median and minimum–maximum. VAS scores were compared between the pre- and postoperative assessments of the operated limb. Plantar flexion, eversion, and SWE values were compared between the operated graft-harvested side and the contralateral healthy side, with comparisons performed using the Wilcoxon signed-rank test. A *p* value of <0.05 was considered to indicate statistical significance.

## Data Availability

Data are contained within the article and Supplementary Materials.

## Supplementary Materials

The following supporting information can be downloaded at https://www.mdpi.com/article/10.3390/jcm15124473/s1, Table S1: Correlation and effect size analyses.
