# Supplementary material for: Assessment of Donor-Site Morbidity Using Shear Wave Elastography After Peroneus Longus Autograft Harvest for ACL Reconstruction
Source: J Clin Med. 2026 Jun 9;15(12):4473. doi: 10.3390/jcm15124473 (PMC13302420; doi:10.3390/jcm15124473)
Supplement: Supplementary file 1 [file jcm-15-04473-s001.zip › jcm-4311385-supplementary.pdf]

Supplementary Table S1. Correlation and effect size analyses.

Analysis	Variables	r	p	Interpretation
Correlation	VAS—SWE (operated)	-0.629	<0.001	Strong negative
Correlation	VAS—SWE (non-operated)	-0.602	<0.001	Strong negative
Correlation	SWE—Plantar flexion	0.336	0.034	Weak–moderate positive
Correlation	SWE—Eversion	-0.083	>0.05	Not significant
Correlation	Plantar flexion (op vs. non-op)	0.899	<0.001	Strong positive
Correlation	Eversion (op vs. non-op)	0.896	<0.001	Strong positive
Correlation	SWE (op vs. non-op)	0.950	<0.001	Very strong positive
Effect size	VAS	0.88	-	Large
Effect size	Plantar flexion	0.02	-	Negligible
Effect size	Eversion	0.10	-	Small
Effect size	SWE	0.18	-	Small

r: Spearman correlation coefficient. $p < 0.05$ was considered statistically significant.
